# Tuberculides

**Published:** 1908-06-27

**Authors:** 


### Dermatology.

### TUBERCULIDES.

The name "tuberculide" was proposed some ten years ago by French writers to designate certain forms of eruptions which have been long known to be associated with tuberculosis, but which are thought to be due, not to the actual presence of the tubercle bacillus in the skin lesion, but to the toxins which are produced by tuberculosis elsewhere in the body. Now that these eruptions, formerly described under various names, have been grouped together, and more closely studied it is found that their occurrence is far from rare. The lesions of these eruptions are characterised by a circumscribed cellular exudation in the corium, which has a certain tendency to break through the epidermis, and thus to form ulcerations with subsequent scar formation. The lesions vary in size from small pin-head follicular papules to filbert-sized nodules. They are usually of one size in any individual case, and from this fact and because they tend to have characteristic groupings and distributions there are several distinct clinical varieties. These varieties may be called (1) small follicular tuberculide, (2) large papular tuberculide, (3) nodular tuberculide, or, better, one may retain the old names of these affections, which were given to them long before they were grouped together as tuberculides. Then we have the following clinical groups: lichen scrofulosorum, acne scrofulosorum, erythema induratum scrofulosorum (or Bazin's disease).

*Lichen Scrofulosorum* is an eruption most often met with in children, although it may occur in adults. The eruption is almost insignificant, but it is of importance because its presence suggests that the patient is suffering from some form of tuberculosis. It may be seen in association with tuberculous glands, with bone or joint tuberculosis, or with tuberculous pleurisy, or there may be no evident manifestation of tubercle. The eruption consists of small, pinhead sized, red follicular papules, arranged in groups or patches of from half an inch to several inches across. These patches are usually situated on the trunk, less often upon the limbs. The eruption may persist for many months, and eventually it dies away, sometimes leaving minute pigmented scars.

*Acne Scrofulosorum.*—Here the lesions are larger papules varying in size from that of a hemp-seed to a small pea. The papules are dusky-red, at first to be felt under the skin, later involving the epidermis, and breaking through to form a central crust. The crust, on falling, leaves a small punched-out ulcer, which cicatrises as a thin circular scar. These lesions are seen mainly about the limbs, particularly upon the thighs and legs. Often the extremities are dusky from impaired circulation. The lesions are also sometimes associated with tuberculous manifestations, but they are often seen when no direct evidence of tuberculosis can be obtained. A variant of this eruption is to be seen (in adults generally) upon the face, while the limbs are comparatively free. This has been given the names of acnitis and acne agminata, but it is probably a form of acne scrofulosorum.

*Erythema Induratum.*—In this affection there are deeply seated, painless, indolent nodules, which occur nearly always upon the legs, and especially upon the calves. The patients are often young servant girls with poor circulation, who have to stand much; these factors probably account for the particular localisation of the lesions. The nodules vary in size from that of a large pea up to that of a filbert nut. At first they are better felt than seen, but soon they reach the surface and form dusky red nodules. They may become absorbed, or they may break down, then forming circular punched-out ulcers, which leave well-marked depressed scars. Here again there is often some sign of tuberculosis, such as enlarged glands; or there may be a strong tuberculous history.

In the treatment of all these affections attention to the general health is the main line to take. The patient should be put into the best possible conditions of fresh air and good food. In hospital patients cod-liver oil is often the only available remedy. Locally, mild mercury ointments are strongly recommended. In nodular tuberculide, or Bazin's disease, rest, with the feet up, is essential.

Many writers in France class lupus erythematosus with the tuberculides. Most observers now agree that it is distinct from lupus vulgaris, and is not due to the tubercle bacillus; but all will not admit that it has a relationship with tuberculosis such as entitles it to be regarded as a tuberculide.

